# Increased response to sequential infections of honeybee, *Apis mellifera scutellata*, colonies by socially parasitic Cape honeybee, *A. m. capensis*, workers

**DOI:** 10.1038/s41598-019-43920-1

**Published:** 2019-05-20

**Authors:** Peter Neumann, Christian W. W. Pirk

**Affiliations:** 1 Institute of Bee Health, Vetsuisse Faculty, University of Bern, Bern, Switzerland; 2 Department of Zoology and Entomology, University of Pretoria, Pretoria, South Africa

**Keywords:** Behavioural ecology, Coevolution

## Abstract

Cape honeybee, *Apis mellifera capensis*, workers can be social parasites and host colonies can defend themselves by rejection of such workers. Using the pseudo-clonal obligate parasitic lineage of *A. m. capensis* and wild-type *A. m. capensis* workers, which are facultative parasites, we show that host colonies significantly increase their defence behaviour towards social parasites upon secondary exposure. Most obligate and facultative social parasites were rejected before they could even produce significant amounts of the queen-like mandibular gland pheromone secretion or activate their ovaries. This suggests that other signals, like cuticular hydrocarbons, could be used by host colonies to identify potential invaders. Honeybee colonies seem to be able to utilise these potential cues, learn from their initial exposure to hive intruders and enable them to improve their defensive behaviour during subsequent infestations, resulting in increased removal rates of parasites.

## Introduction

Cape honeybee, *Apis mellifera capensis* Eschscholtz, workers can be social parasites in colonies of their own and other subspecies, such as the neighbouring one, *Apis mellifera scutellata* Lepeletier^1–8^. After successful invasion, socially parasitic workers often develop a pseudoqueen phenotype^9^ including the activation of ovaries and the production of a queen-like pheromonal bouquet^10^. These workers parthenogenetically produce female offspring via thelytoky^11^. In the course of infestation, the host queen is lost^7^. The colonies are then gradually taken over and eventually die^7,9^.

Since 1990, socially parasitic *A. m. capensis* workers have invaded *A. m. scutellata* colonies on a large scale, resulting in the loss of thousands of colonies per year^6,7,12^. As there is high genetic variance for reproductive dominance in *A. m. capensis*^9,13^ selection for the most dominant socially parasitic worker genotype is favoured within the *A. m. scutellata* host population. In fact, the pseudo-clonal offspring of a single parasitic worker^11,14,15^ have caused infestations in the north-eastern parts of South Africa and is now covering an area of approximately 275.000 km^2,14^. All parasitic workers in the range of *A. m. scutellata* bear the genetic signature of a clone founded by a single ancestral worker genotype^11,14,15^.

Despite this “*capensis* calamity”^12^, *A. m. scutellata* colonies have some resistance, because host workers can show rejection behaviour, e.g. attack and eject parasitic workers^9^. Previous studies on parasite infection have shown that honeybee colonies may eject small hive beetle invaders more quickly after a second exposure^16^. This might also be the case for socially parasitic workers and could be adaptive given high chances of multiple infections. Moreover, identification might be specific to certain types of parasites even within a subspecies. To test if honeybee workers have the ability to identify specific, even closely related, parasites, we took advantage of the pseudo-clonal socially parasitic worker lineage of the Cape honeybee, *A. m. capensis*^7,11,17^. If honeybee colonies are able to recognize this lineage specifically, we would expect them to remove such parasites more quickly compared to other honeybee lineages and subspecies.

## Results

**Figure 1:**
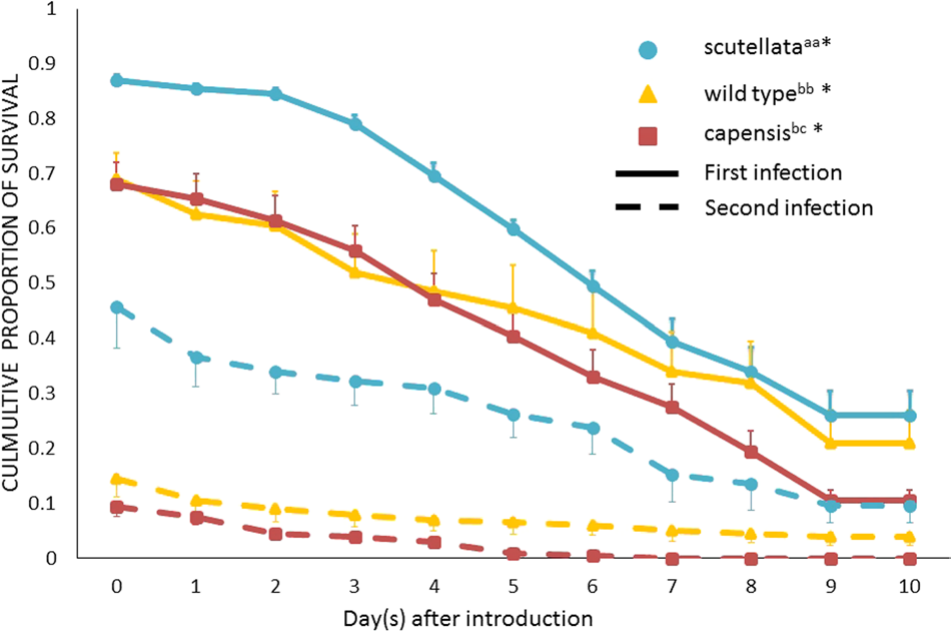
Proportion of control, obligate and facultative parasitic workers surviving per day after sequential infections. Comparison of the survival of the three different worker groups in each infection using Cox-Mantel tests. Mean and SE of the cumulative proportion of survival by day of the 1^st^ and 2^nd^ infection (36 h later) are shown. First letter, if different, indicates significant differences in survival between different worker groups within first infection. Second letter, if different, indicates significant differences in survival between different worker groups within second infection. Significant differences between the sequential infections within one worker group are indicated by asterisks.

The survival analysis revealed that during both infections the parasitic pseudo-clones were identified and removed significantly faster than *A. m. scutellata* control (Fig. 1; pseudo-clone vs. *scutellata* control: Infestation 1 Test statistics (TS) = 4.74 p < 0.00001; Infestation 2 TS = 7.58 p < 0.00001). Only during the second infestation were the parasitic pseudo-clones identified and removed significantly faster than *A. m. capensis* non-clonal social parasites (Fig. 1; pseudo-clone vs. *A. m. capensis* non-clonal: Infestation 1 TS = 2.23 n.s.; Infestation 2 TS = 2.7 p < 0.0068). Furthermore, *A. m. scutellata* control workers were removed significantly more slowly by the host colonies than *A. m. capensis* non-clonal social parasites (*scutellata* vs. non-clonal parasites: TS > 2.32, p < 0.02 for infection 1 and 2). For each individual test group, individuals of the same group were removed at a faster rate from the test colonies during the second encounter as compared to the first infections (TS > 8.45, p < 0.00001, for all three pair wise comparisons). By comparing the increase in the average daily mortality from the first to the second round of infections it was revealed that the pseudo-clonal group increased from an average mortality of 19.7% to 39.4% (z = 4.31, p < 0.0001) during the second infestation, the *A. m. scutellata* control group increased from 13.8% average daily mortality to 21.3% (z = 1.66, n.s.) and the non-clonal parasitic group from 16.8% to 31.4% (z = 3.38, p < 0.001).

## Discussion

The data clearly show that both, the socially parasitic pseudo-clonal and the non-clonal social parasites *A. m. capensis* workers were rejected significantly faster upon secondary exposure.

Pheromonal competition governs reproductive dominance hierarchies among *A. m. capensis* workers^18,19^. The tested clonal parasitic worker lineage has probably been selected for rapid queen-like mandibular gland secretions^20,21^ as well as for its resistance against host queen pheromone signals^19,22^. The observed identification and rejection of socially parasitic workers during the first infection by the host workers is therefore most likely related to the faster reproductive development of the pseudo-clonal lineage^23^, similar to aggressive behaviour shown by non-developed workers towards workers with ongoing reproductive development in other subspecies^24^. Furthermore, internal anatomical differences like spermatheca size and numbers of ovarioles^25^ could result in differential volatile profiles. Pheromones could be another set of factors, in particular the pheromonal predisposition of *A. m. capensis* workers seen even under queenright conditions^26^ and the fact that the gland secretion of the parasitic lineage is significantly different to subordinate workers^27^. These differences in the gland and cuticular profile would allow the host workers to learn parasite specific cues, which allows them to identify and remove them in a subsequent infestation^26,27^.

Furthermore, the results show that during the second encounter the pseudo-clonal parasitic workers are rejected at a much faster rate by the *A. m. scutellata* host workers than all other tested groups. That pseudo-clonal parasitic workers are rejected faster could either be because of the genetically homogeneity of the lineage, which could make it is easier to learn its specific cues for the host or it is indicating a specifically directed behavioural response. More than 90% of the pseudo-clonal parasitic workers were rejected in less than 24 hours (Fig. 1) and the faster removal of the non-clonal social parasites suggests that it is rather a specifically directed behavioural response. This would mean that the faster the behavioural reaction, the stronger the resulting protection of the host colony as *A. m. capensis* workers need on average 6.5 days to become reproductively active^23^. Indeed, the callow parasitic workers did not remain long enough in the host colonies to activate their ovaries^23^, but signals from the tergal gland and cuticle could be used to discriminate between hosts and parasites^19,26^. Therefore, the parasitic workers could not establish themselves as reproductive parasites and can be excluded before they are able to gain reproductive dominance^9,26^ and before they are able to overthrow colony defences. In any case, the observed fast behavioural reaction of the host workers upon secondary exposure provides strong support to earlier findings for the ejection of small hive beetle parasites by honeybee colonies^16^. Taken these observations together, it appears as if honeybee colonies may be able to learn from their initial exposure to hive intruders and are able to improve their defensive behaviour, resulting in increased removal rates of parasites. The observation that the host colonies are able to evict social parasitic workers more swiftly during the second encounter could either suggest that the host colonies get conditioned or that honeybees have evolved a potential social immune memory to fight conspecific parasites. Both are not mutually exclusive and would result in the same advantageous behaviour of repelling potential social parasites.

## Material and Methods

### Study animals and experimental set up

Queenright, unrelated *A. m. scutellata* colonies (N = 4) were obtained from its endemic range (Pretoria, Gauteng Province, South Africa). All of them were set up in three-frame observation hives (∼3,000 bees). The middle frame in each hive contained brood and the top and bottom frames honey and pollen. Colonies were fed *ad libitum* with sugar water (1:1) and artificial beebread (honey/icing sugar/soya flour 1:2:2). On the same day of establishing the observation hives, frames with sealed worker brood were placed in an incubator until adult emergence^28^. Twenty-four hours later, we introduced three different groups of freshly emerged individually labelled test workers (N = 50 each group, <24 h old) into the four queenright test colonies:

1. *A. m. capensis* social parasites with diverse genotypes (facultative parasitic lineage): offspring of a naturally mated queen from the endemic range of the Cape honeybee (Heidelberg, Western Cape).
2. *A. m. capensis* social parasites with pseudo-clonal genotypes (obligate parasitic lineage): thelytokous offspring of the socially parasitic *A. m. capensis* worker lineage^11,14,29^ from a heavily infected queenless *A. m. scutellata* host colony (Pretoria, Gauteng^30^).
3. *A. m. scutellata* host workers: offspring of a naturally mated queen from Pretoria (Gauteng) unrelated to the recipient colonies.

The introduction of all 150 workers took less than five minutes and freshly emerged workers were used because they are normally readily accepted^31^ as the colony odour in honeybees is strongly affected by environmental cues^32^. The experiments were conducted in 2004, all workers were labelled individually with Opalithplättchen (numbered plastic tags) and observation hives were screened for the presence of the introduced workers twice a day (13:00 day light and 18:00 red light conditions) following previously used methods^33^.

### Procedure

All observation hives were infected at the same time and screened twice daily for the presence of labelled workers for 10 days and after the period, all test workers were carefully removed. The infections were repeated with new test workers 36 h thereafter using the same colonies and protocols.

### Data analyses

The data were analysed using Cox-Mantel survival analysis. Pair-wise comparisons were performed between the different groups and the Bonferroni adjustment was applied. The data are displayed as the cumulative proportion surviving. For the Cox-Mantel analysis both the test statistic (TS) and p values are given. To evaluate if any of the groups received a stronger behavioural rejection during the second round of infection we calculated the overall average daily mortality and statistically compared the proportions. All statistical analyses were performed using Statistica^©^.
